# A Bacteriophage Tailspike Domain Promotes Self-Cleavage of a Human Membrane-Bound Transcription Factor, the Myelin Regulatory Factor MYRF

**DOI:** 10.1371/journal.pbio.1001624

**Published:** 2013-08-13

**Authors:** Zhihua Li, Yungki Park, Edward M. Marcotte

**Affiliations:** Center for Systems and Synthetic Biology, Institute for Cellular and Molecular Biology, The University of Texas at Austin, Austin, Texas, United States of America; University of Edinburgh, United Kingdom

## Abstract

Myelination of the central nervous system (CNS) is critical to vertebrate nervous systems for efficient neural signaling. CNS myelination occurs as oligodendrocytes terminally differentiate, a process regulated in part by the myelin regulatory factor, MYRF. Using bioinformatics and extensive biochemical and functional assays, we find that MYRF is generated as an integral membrane protein that must be processed to release its transcription factor domain from the membrane. In contrast to most membrane-bound transcription factors, MYRF proteolysis seems constitutive and independent of cell- and tissue-type, as we demonstrate by reconstitution in *E. coli* and yeast. The apparent absence of physiological cues raises the question as to how and why MYRF is processed. By using computational methods capable of recognizing extremely divergent sequence homology, we identified a MYRF protein domain distantly related to bacteriophage tailspike proteins. Although occurring in otherwise unrelated proteins, the phage domains are known to chaperone the tailspike proteins' trimerization and auto-cleavage, raising the hypothesis that the MYRF domain might contribute to a novel activation method for a membrane-bound transcription factor. We find that the MYRF domain indeed serves as an intramolecular chaperone that facilitates MYRF trimerization and proteolysis. Functional assays confirm that the chaperone domain-mediated auto-proteolysis is essential both for MYRF's transcriptional activity and its ability to promote oligodendrocyte maturation. This work thus reveals a previously unknown key step in CNS myelination. These data also reconcile conflicting observations of this protein family, different members of which have been identified as transmembrane or nuclear proteins. Finally, our data illustrate a remarkable evolutionary repurposing between bacteriophages and eukaryotes, with a chaperone domain capable of catalyzing trimerization-dependent auto-proteolysis in two entirely distinct protein and cellular contexts, in one case participating in bacteriophage tailspike maturation and in the other activating a key transcription factor for CNS myelination.

## Author Summary

Membrane-bound transcription factors are synthesized as integral membrane proteins, but are proteolytically cleaved in response to relevant cues, untethering their transcription factor domains from the membrane to control gene expression in the nucleus. Here, we find that the myelin regulatory factor MYRF, a major transcriptional regulator of oligodendrocyte differentiation and central nervous system myelination, is also a membrane-bound transcription factor. In marked contrast to most well-known membrane-bound transcription factors, cleavage of MYRF appears to be unconditional. Surprisingly, this processing is performed by a protein domain shared with bacteriophages in otherwise unrelated proteins, where the domain is critical to the folding and proteolytic maturation of virus tailspikes. In addition to revealing a previously unknown key step in central nervous system myelination, this work also illustrates a remarkable example of evolutionary repurposing between bacteriophages and eukaryotes, with the same protein domain capable of catalyzing trimerization-dependent auto-proteolysis in two completely distinct protein and cellular contexts.

## Introduction

Membrane-bound transcription factors (MBTFs) are a remarkable class of transcription factors that are initially generated as integral membrane proteins. Upon relevant cues, they undergo proteolytic processing, releasing the transcription factor domain from the membrane and allowing it to translocate to the nucleus to control gene expression. Two different broad mechanisms of MBTF proteolytic activation have been observed to date. One class of MBTFs is proteolytically activated by regulated ubiquitin/proteasome-dependent processing (RUP) and includes transcription factors that control membrane fluidity in budding yeast (SPT23 and MGA2) and a fission yeast hypoxic transcription factor (Sre1) [1]–[2]. The second class is activated *via* regulated intramembrane proteolysis (RIP) and includes sterol regulatory element-binding proteins (SREBPs) [3]–[4], activating transcription factor 6 (ATF6) [5]–[7], and the developmental regulator Notch [8]–[10]. RIP-dependent activation of MBTFs typically requires additional proteases that act outside of the membrane. For example, when cellular cholesterol levels decrease, SREBPs are transported to the Golgi apparatus, where they are cleaved by Site-1 protease, whose active site is located in the lumen of the Golgi. Cleavage by Site-1 protease allows the subsequent intramembrane proteolysis by Site-2 protease [4]. Similarly, following accumulation of misfolded proteins in the endoplasmic reticulum (ER), ATF6 translocates to the Golgi and is proteolyzed sequentially by Site-1 and Site-2 proteases [5],[7]. Recently, many basic leucine zipper proteins homologous to ATF6 have been discovered and appear to play important roles in tissue-specific unfolded protein responses [11]–[12].

Within the human genome, an early genome-wide computational screen suggested the existence of six MBTFs [13]. Since then, the number of characterized DNA-binding domains has increased significantly [14], and prediction methods for the membrane topology of proteins have been improved dramatically [15]–[16], which led us to revisit the search for human MBTFs. We found that C11orf9, the largely uncharacterized human ortholog of mouse Myrf (a key transcriptional regulator of oligodendrocyte (OL) maturation and CNS myelination [17]), was strongly predicted to encode an MBTF. C11orf9 (hereafter referred to as MYRF [CCDS ID: 31579 and RefSeq ID: NP_037411]) and its orthologs were predicted to have a domain homologous to the DNA-binding domain of the yeast transcription factor Ndt80 [18] as well as a single transmembrane (TM) segment.

However, by using algorithms capable of recognizing extremely distant sequence homology, we also observed that MYRF and its orthologs harbor an intramolecular chaperone domain shared with bacteriophage endosialidases [19]–[20], the tailspike proteins essential for bacteriophages to infect bacteria encapsulated with polysaccharides. While the homology of genes between bacteriophages and eukaryotes is not unprecedented, or even the horizontal transfer of genes between the two, it is nonetheless rare, and in general the mechanism of transferred genes is quite different. For example, the GG domain is found in both bacteriophage tail fibers and FAM3 cytokines [21]. In addition, the large nuclear and cytoplasmic viruses, such as Mimivirus, appear to have chimeric origins that include bacteriophages [22].

The tailspike proteins are known to trimerize and to self-process. This raised the hypothesis that this domain in eukaryotes might contribute to a novel method for the formation and function of an MBTF. Indeed, the intramolecular chaperone domain of MYRF facilitates its homo-oligomerization and proteolytically processes it into two halves. The N-terminal trimer, containing the DNA-binding domain, is released from the ER membrane and moves to the nucleus, where it exerts transcriptional effects. Proper processing and translocation of the MYRF N-terminal trimer then contributes to the maturation of OLs. The C-terminal homo-oligomer, containing the TM domain, remains in the ER. These findings not only demonstrate an extraordinary link between a possible endosymbiont or commensal bacteriophage and eukaryotic development, but reveal a novel cleavage mechanism for MBTFs.

## Results

### Full-Length MYRF Is First Generated as a Type-II Membrane Protein

**Figure 1:**
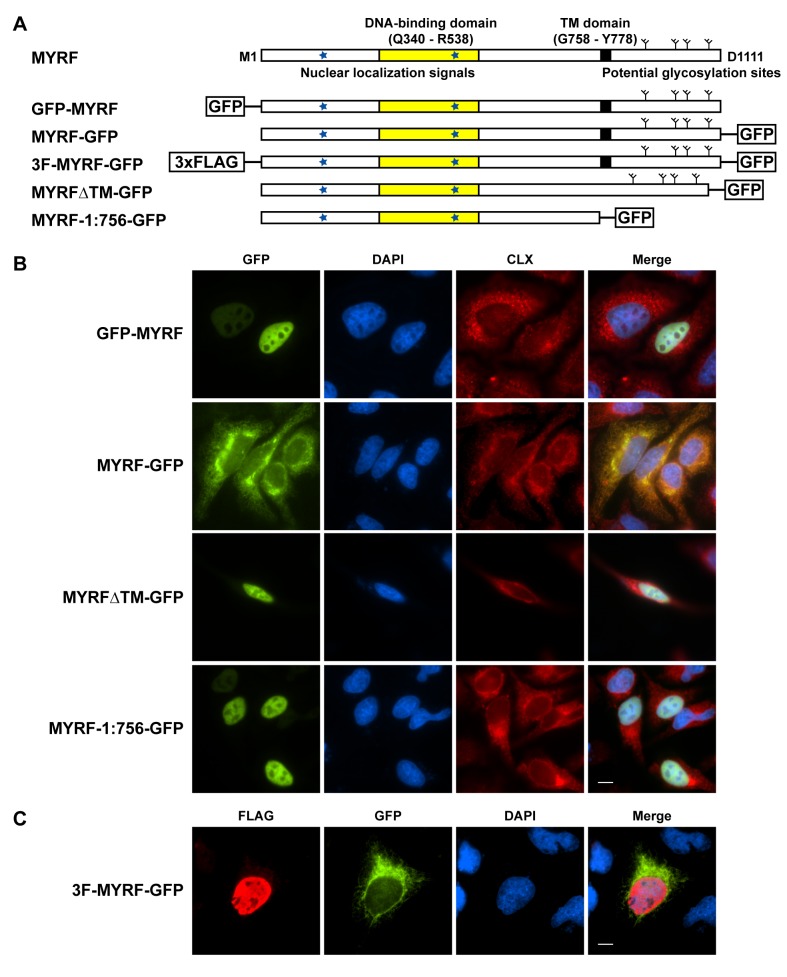
Full-length MYRF is generated as a membrane protein. (A) Predicted sequence features of MYRF and sequence diagrams of various MYRF constructs used for IF microscopy. Stars in blue indicate predicted NLSs at K_245_KRK_248_ and K_482_KGK_485_. (B) IF images of GFP-MYRF, MYRF-GFP, MYRFΔTM-GFP, and MYRF-1:756-GFP in HeLa cells. (C) IF image of 3F-MYRF-GFP in HeLa cells. Scale bar, 10 µm.

Myrf (the mouse ortholog of MYRF) was previously reported to encode a nuclear protein, based on immunofluorescence (IF) microscopy with an N-terminally Myc-tagged construct [17]. However, TOPCONS [15], a state-of-the-art membrane topology prediction program, predicts both MYRF and Myrf to be type-II membrane proteins (Figure S1A). Notably, we identified well-conserved nuclear localization signals (NLSs) in the N-terminus (K_245_KRK_248_ and K_482_KGK_485_) and potential N-linked glycosylation sites in the C-terminus (Figure 1A).

To determine the precise localization of MYRF in the cell, we expressed epitope-tagged MYRF constructs in HeLa cells. Green fluorescent protein (GFP) tagged to the N-terminus of MYRF (GFP-MYRF, Figure 1A) localized to the nucleus, in agreement with the previous study on Myrf (Figure 1B). However, when GFP was tagged to the C-terminus of MYRF (MYRF-GFP, Figure 1A), the GFP signal co-localized with calnexin (CLX), an ER marker (Figure 1B). A doubly-tagged protein, 3F-MYRF-GFP (Figure 1A), resolved this apparent dichotomy: The FLAG tag at the N-terminus exhibited a nuclear signal, whereas the C-terminal GFP signal co-localized with the ER (Figure 1C).

In order to test if the predicted TM domain mediated the ER localization of the C-terminus of MYRF, we deleted the TM domain from the C-terminally GFP-tagged construct (MYRFΔTM-GFP, Figure 1A). MYRFΔTM-GFP localized to the nucleus of HeLa cells (Figure 1B), confirming the role of the predicted TM domain for ER localization. Similarly, a C-terminally GFP-tagged mutant truncated before the predicted TM domain at L756 (MYRF-1:756-GFP, Figure 1A) also localized to the nucleus (Figure 1B). Control experiments were consistent when using alternate epitope tags (FLAG tag; Figure S1B) and cell lines (CG4 cells, a rat OL cell line that may be used as a model for early OL differentiation [23]–[31]; Figure S1C and S1D). Thus, these localization patterns appear to be intrinsic features of MYRF and not artifacts of the particular tags or cells used.

**Figure 2:**
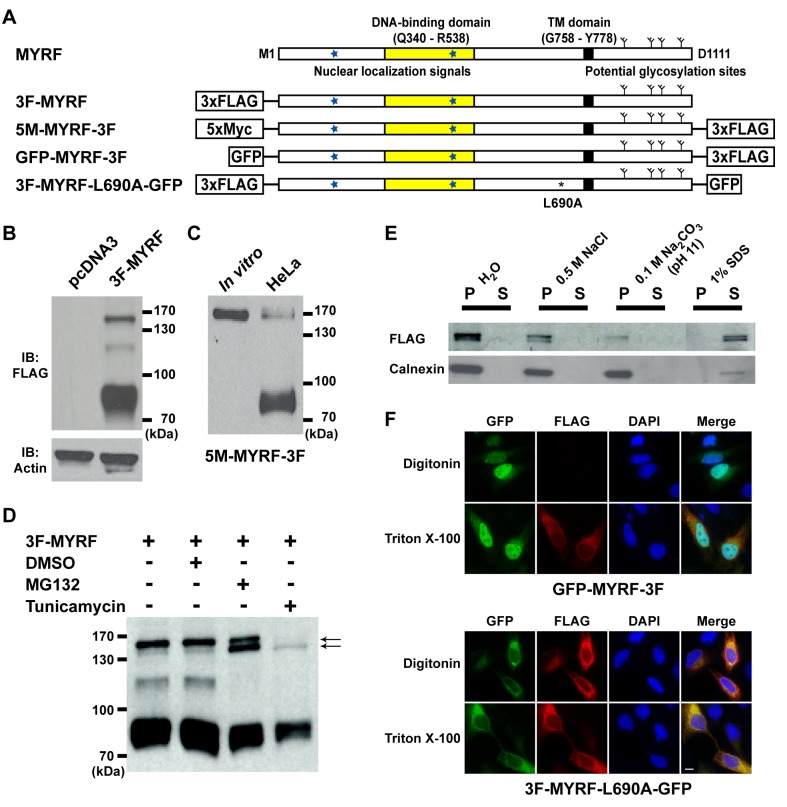
Full-length MYRF is a type-II membrane protein. (A) Predicted sequence features of MYRF and sequence diagrams of various MYRF constructs used for experiments. (B) Western blot of HeLa cells transfected with pcDNA3 and 3F-MYRF. (C) The top band of HeLa cells that were transfected with 5M-MYRF-3F has the same electrophoretic mobility as full-length protein products for the same construct that were obtained with an *in vitro* translation system. (D) Full-length forms of MYRF consist of two closely spaced bands that represent glycosylated and unglycosylated full-length MYRF, respectively (indicated by the two arrows). (E) HeLa cells transfected with 3F-MYRF were disrupted using a Dounce-type homogenizer, and then centrifuged at 200× *g* for 5 min to obtain a supernatant fraction. It was mixed with 0.1 volume of each of the following chemicals: 5 M NaCl, 1 M Na_2_CO_3_ (pH 11), and 10% SDS. After incubation for 20 min at room temperature, mixtures were centrifuged at 20,000× *g* for 15 min at 4°C to separate supernatant (S) from pellet (P). Calnexin, a known integral membrane protein, served as a control. (F) Membrane topology of GFP-MYRF-3F and 3F-MYRF-L690A-GFP in HeLa cells. When cell membranes were selectively permeated by digitonin, FLAG IF signals of GFP-MYRF-3F could not be detected, indicating that the C-terminus of MYRF is located within the ER lumen. In contrast, FLAG IF signals of 3F-MYRF-L690A-GFP were robustly detected even when cell membranes were selectively permeated by digitonin, indicating that the N-terminus of full-length MYRF is located on the cytoplasmic side of ER membranes. Scale bar, 10 µm.

The microscopy suggested that MYRF is processed in cells, which was further confirmed by Western blot of 3F-MYRF (Figure 2B). The majority of the protein was cleaved into a ∼90 kDa N-terminal fragment from the full length of ∼160 kDa. The latter was further verified by comparing 5M-MYRF-3F protein expressed in cells to that expressed from an *in vitro* translation system (the *in vitro* reaction mixture immunoprecipitated with FLAG antibodies and blotted with anti-Myc antibodies) (Figure 2C).

The top band representing full-length MYRF was observed to consist of two closely spaced bands (Figure 2D, arrows), with the upper and lower bands potentially representing glycosylated and unglycosylated full-length MYRF, respectively. Upon MG132 treatment, the lower band became as dominant as the upper one. This suggested either that MG132 treatment alters the degradation of MYRF or that MG132—an inducer of ER stress that decreases glycosylation efficiency [32]—inhibits the glycosylation of full-length MYRF, leading to the accumulation of unglycosylated full-length MYRF. Consistent with the latter possibility, tunicamycin treatment reversed the ratio between the upper and lower bands, with the lower one now dominating (Figure 2D). We note that the 120 kDa isoform (Figure 2B) is most likely a degradation intermediate, as it was inconsistently observed and disappeared upon treatment with MG132 (Figure S2A).

Fractionation of HeLa cells transfected with 3F-MYRF revealed that full-length MYRF could be extracted from membranes by treatment with the detergent SDS, but not with high salt or alkaline pH (Figure 2E), similar to the control protein calnexin, a known integral membrane protein. Thus, the fluorescence microscopy, TM domain mutagenesis, glycosylation analysis, and biochemical fractionation data all demonstrated that full-length MYRF is an integral membrane protein.

Finally, we determined the membrane topology of full-length MYRF by treating cells with digitonin, which selectively permeabilizes the plasma membrane but not organelle membranes (Figure S2B) [33]. When the plasma membrane of HeLa cells expressing GFP-MYRF-3F was selectively permeabilized by digitonin, FLAG IF signals could not be detected, in contrast to a strong signal when membranes were indiscriminately permeabilized by Triton X-100 (Figure 2F), suggesting that the C-terminus of MYRF is oriented to the ER lumen. Additional tests with a point mutant (L690A, detailed below) that blocks the generation of the 90 kDa isoform from full-length MYRF enabled us to probe the subcellular location of the N-terminus of full-length MYRF. FLAG IF signals were detected for 3F-MYRF-L690A-GFP when cell membranes were selectively permeated with digitonin (Figure 2F), indicating that the N-terminus of full-length MYRF is located on the cytoplasmic side of ER membranes. Thus, MYRF is synthesized as a type-II membrane protein and processed into N-terminal and C-terminal portions, localized in the nucleus and on the ER membrane, respectively.

### MYRF Harbors the Intramolecular Chaperone Domain of Bacteriophage Endosialidases

In the course of analyzing the MYRF sequence, we discovered distant but significant homology (16% sequence identity and *E*-value = 3.1×10^−18^, as measured by HHpred [34]) between the portion of MYRF that lies between its DNA-binding and TM domains and the intramolecular chaperone domain found in bacteriophage endosialidases, proteins that constitute the tailspikes of many bacteriophages (Figure S3A) [19],[35]. The intramolecular chaperone domain, which we have dubbed an ICA (Intramolecular Chaperone Auto-processing) domain, plays two roles in the maturation of bacteriophage endosialidases. The ICA domain facilitates the protein's folding and trimerization [19],[35]. It then functions as a “folding sensor” and auto-cleaves itself away from the bacteriophage endosialidase [20].

**Figure 3:**
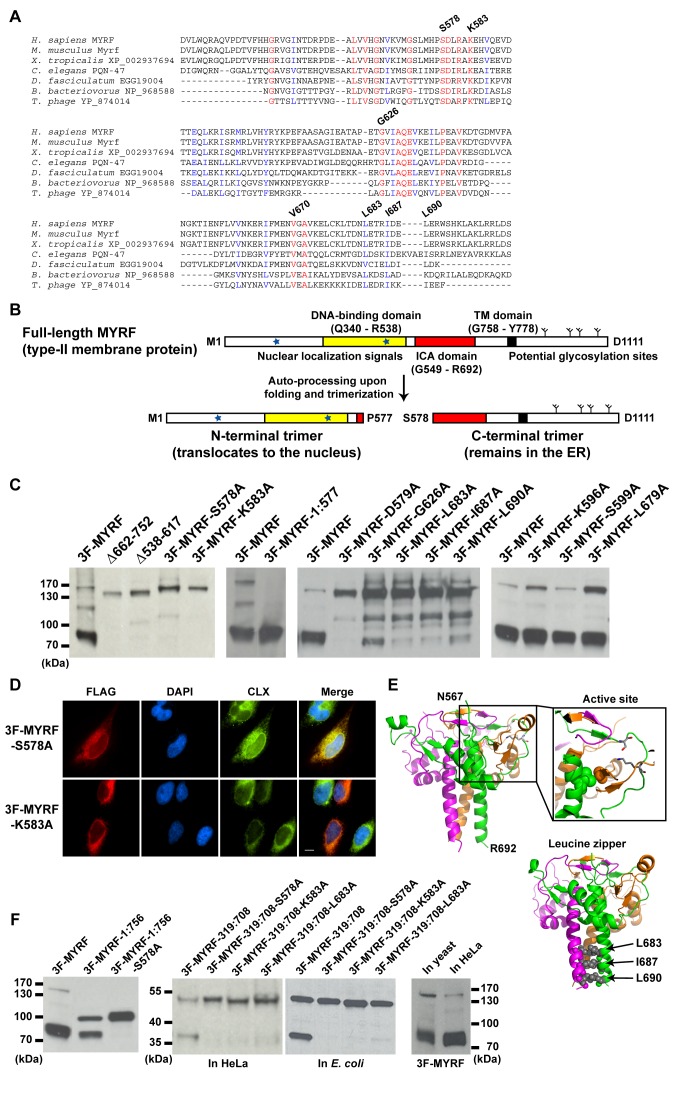
The ICA domain autonomously mediates the proteolytic processing of MYRF. (A) Multiple sequence alignment of the ICA domains from eukaryotes, a bacterium, and a phage, generated with ClustalW [54]. Strictly conserved residues are shown in red. The numbering system is based on MYRF. (B) The auto-processing mechanism for MYRF postulated based on the ICA domain and its known properties. (C) Western blots of HeLa cells transfected with various MYRF constructs, showing the effects of mutations in the ICA domain on the proteolytic processing of MYRF. (D) IF image of 3F-MYRF-S578A and 3F-MYRF-K583A in HeLa cells. (E) The amino acid sequence of MYRF (residues N567-R692) was mapped onto the crystal structure of an ICA domain (PDB ID: 3GW6) using the alignment shown in Figure S3A. In the zoomed active site are shown two key catalytic residues (S578 and K583, both belonging to the same subunit) in stick model and two strictly conserved residues (V670 of one subunit and G626 of a different subunit) in space filling model. Shown below are L683, I687, and L690 that were predicted to form a leucine zipper. For visual clarity, clipped images were generated when deemed necessary. (F) (Left) Western blot showing that the proteolytic processing of MYRF is independent of its membrane insertion. MYRF-1:756 is a mutant truncated before the TM domain at L756. (Middle) Western blot showing the proteolytic processing of MYRF-319:708 in HeLa cells and *E. coli*. (Right) Western blot showing the normal processing of full-length MYRF in budding yeast. Scale bar, 10 µm.

A multiple sequence alignment of MYRF and its orthologs indicated that the ICA domain is a strictly conserved feature (Figure S3D). Further, a multiple sequence alignment of only the ICA domains from eukaryotes, a bacterium, and a phage revealed the absolute conservation of S578 and K583 (following the MYRF numbering, Figure 3A). In bacteriophage endosialidases, the serine and lysine residues equivalent to MYRF S578 and K583 form a catalytic dyad for the auto-cleavage reaction [20]. The correct positioning of these catalytic residues, along with an arginine residue that stabilizes the oxyanion during the peptide bond breakage, is thought to be achieved only upon folding and trimerization of bacteriophage endosialidases [20], enabling the ICA domain to function as a folding sensor. We thus asked if the ICA domain might nonetheless still serve—in a radically altered context as compared to viral tailspikes—as a folding sensor and protease to activate MYRF.

### The ICA Domain Autonomously Mediates the Proteolytic Processing of Full-Length MYRF

Based on the conservation of the ICA domain, including its catalytic residues, we hypothesized that, once generated as a type-II membrane protein, the ICA domain could potentially facilitate the folding and trimerization of full-length MYRF and then proteolytically process it into two independent trimers (Figure 3B). The N-terminal trimer, containing the DNA-binding domain, might then be released from membranes and enter the nucleus to regulate transcription, while the C-terminal trimer, comprising residues S578-D1111, would remain in the ER membrane.

To test this hypothesis, we mutated the ICA domain of MYRF and assayed the effects on the proteolytic processing of MYRF. Deletions involving the ICA domain (Δ662–752 and Δ538–617) blocked normal processing of MYRF (Figure 3C). Likewise, mutation of the putative catalytic residues S578 and K583 to alanine (3F-MYRF-S578A and 3F-MYRF-K583A) also blocked proteolytic processing (Figure 3C). The FLAG tag at the N-terminus of these mutant constructs remained in the ER membrane of HeLa cells (Figure 3D), demonstrating that the DNA-binding domain of MYRF is retained in the membrane when auto-processing is blocked.

We next asked whether additional residues shown to be important for the function of phage ICA domains are also important for the function of the MYRF ICA domain. As the N912 and G956 residues in the ICA domain of bacteriophage K1F endosialidase are essential for the function of the ICA domain [19], mutation of their corresponding residues in MYRF to alanine (3F-MYRF-D579A and 3F-MYRF-G626A) markedly reduced the proteolytic processing of MYRF (Figure 3C). As an additional control, we expressed a truncated form of MYRF that terminates at P577 (3F-MYRF-1:577), corresponding to the expected N-terminal fragment generated from auto-processing (Figure 3B), and confirmed that it has the same electrophoretic mobility as the processed N-terminal fragment of 3F-MYRF (Figure 3C). Taken together, these results support the hypothesis that the ICA domain mediates the proteolytic processing of MYRF, in a manner similar to bacteriophage endosialidases.

To further investigate the role of the ICA domain in the processing of MYRF, we mapped the amino acid sequence of the MYRF ICA domain (residues N567–R692) onto the crystal structure of the ICA domain of bacteriophage K1F endosialidase (PDB accession code: 3GW6 [20]) (Figure S3A). The homology-derived structure predicted that L683, I687, and L690 of MYRF form a leucine zipper (Figure 3E). Because the leucine zipper appears integral to the trimeric structure of the MYRF ICA domain, we reasoned that its disruption would destabilize the trimer and consequently interfere with proteolytic processing. Site-directed mutagenesis confirmed that the leucine zipper is indeed required for MYRF processing (Figure 3C). IF microscopy of 3F-MYRF-L683A and 3F-MYRF-L690A in HeLa cells confirmed that their localizations matched the catalytic residue mutants (Figure S3B). In contrast, the structure suggested that K596, S599, and L679 would not be essential to either catalytic or structural roles, and all were predicted to face the exterior of the protein (Figure S3C). Consistent with this prediction, mutating each of these residues to alanine (3F-MYRF-K596A, 3F-MYRF-S599A, and 3F-MYRF-L679A) did not affect MYRF processing (Figure 3C). These results confirm that the ICA domain is indeed responsible for the proteolytic processing of MYRF, and that the mechanism of proteolysis is conserved between animals and bacteriophages, in spite of a complete alteration of neighboring protein domains and overall protein function.

The ICA domain is known to function autonomously to proteolyze bacteriophage endosialidases. We therefore asked whether the processing of MYRF was similarly autonomous, testing two specific hypotheses. First, we examined whether the proteolytic processing of MYRF was independent of membrane integration. As shown in Figure 3F, a construct (3F-MYRF-1:756) that was truncated before the TM domain at L756 was normally processed in HeLa cells, but processing was blocked when the catalytic residue S578 was changed to alanine (3F-MYRF-1:756-S578A). Second, we asked whether MYRF is normally processed in heterologous systems, which would support a fully autonomous event. To address this hypothesis, we expressed MYRF in *E. coli* and yeast cells. Due to the difficulty of expressing full-length MYRF in *E. coli*, we worked with a truncation construct (MYRF-319:708) that only comprises the DNA-binding and ICA domains of MYRF. This construct was normally processed in HeLa cells (Figure 3F), and its processing was blocked when important residues were mutated to alanine (3F-MYRF-319:708-S578A, 3F-MYRF-319:708-K583A, and 3F-MYRF-319:708-L683A). Figure 3F shows that MYRF-319:708 behaved in the same manner in *E. coli*, and similarly, full-length MYRF was normally processed in budding yeast (Figure 3F). Taken together, these results indicate that the ICA domain autonomously functions in the proteolytic processing of MYRF.

### The N-Terminal Trimer, Formed by the ICA Domain, Translocates to the Nucleus Aided by Two NLSs

**Figure 4:**
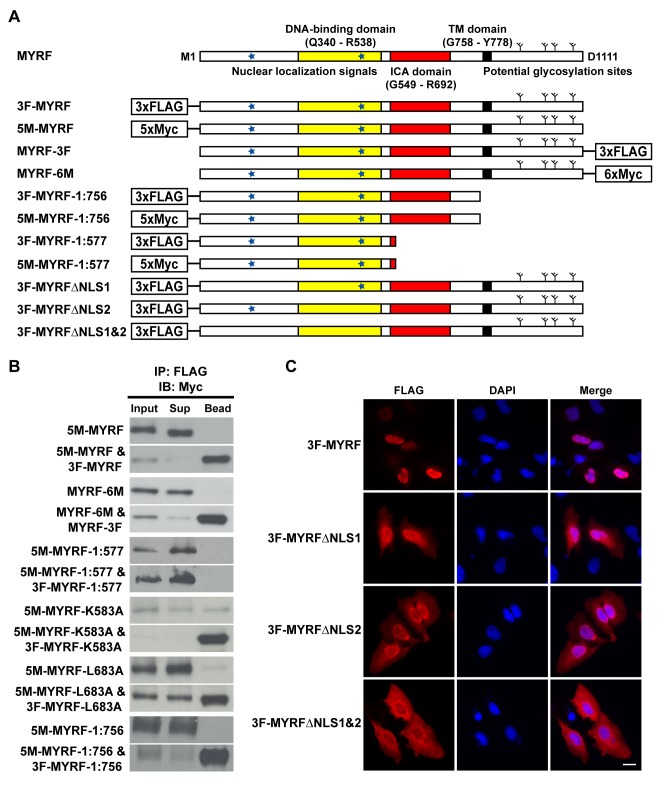
The N-terminal trimer is formed by the ICA domain and enters the nucleus. (A) Predicted sequence features of MYRF and sequence diagrams of various MYRF constructs used for experiments. (B) Western blots showing co-immunoprecipitation results for the MYRF constructs. “Input” was incubated with FLAG antibody-coated beads and then spun down to separate “Sup” from “Bead” fractions. The failure of MYRF-1:577 to homo-oligomerize demonstrated the importance of the ICA domain for the N-terminal trimer formation. (C) When the NLSs (NLS1 and NLS2) were deleted, the nuclear translocation of the N-terminal trimer was partially blocked. Scale bar, 20 µm.

The ICA domain is known to induce the trimerization of bacteriophage endosialidases as part of its intramolecular chaperone activity [36]. Given the central role of the ICA domain in MYRF auto-processing, we next asked whether it was also promoting trimerization in this context. We first used co-immunoprecipitation experiments of differentially tagged constructs in order to assay homo-oligomerization of the N-terminal fragment generated by the auto-processing of MYRF. As shown in Figure 4B, the N-terminal fragment of N-terminally 5xMyc-tagged MYRF (5M-MYRF; Figure 4A) did not bind beads coated with FLAG antibodies. However, when co-transfected with 3F-MYRF (Figure 4A), the N-terminal fragment of 5M-MYRF robustly bound the FLAG beads, confirming homo-oligomerization of the N-terminal fragment of MYRF. To measure the nature of the homo-oligomer, we employed size exclusion chromatography, which indicated that the N-terminal fragment from the auto-processing of MYRF-319:708 (a construct which only contains the DNA-binding and ICA domains of MYRF) forms a trimer (Figure S4). MrfA, a *Dictyostelium* ortholog of MYRF, has also been suggested to bind DNA as a trimer *in vivo* [37].

Given that the MYRF N-terminal fragment forms a trimer, we assayed the state of the C-terminal fragment. We could not directly assay its trimeric state due to expression and purification issues in *E. coli*; instead, we asked if the C-terminal fragment of MYRF also homo-oligomerized using co-immunoprecipitation. As predicted, the C-terminal fragment of MYRF-6M (Figure 4A) did not bind FLAG beads (Figure 4B). However, co-transfection with MYRF-3F (Figure 4A) induced binding (Figure 4B), confirming that the C-terminal fragment generated from auto-processing is also a homo-oligomer. Because the bacteriophage ICA domain is known to exist autonomously as a trimer [19]–[20] and the ICA domain is part of the C-terminal fragment of MYRF, and we have confirmed the trimeric state of the N-terminal fragment, we expect the MYRF C-terminal fragment to also exist as a trimer.

Because the ICA domain is known to induce trimerization, we expected that a truncated construct encoding only the N-terminal fragment (MYRF-1:577; Figure 4A) should fail to trimerize. When expressed alone in HeLa cells, 5M-MYRF-1:577 did not bind FLAG beads (Figure 4B). When co-transfected with 3F-MYRF-1:577, it still did not bind (Figure 4B), even though co-transfected 3F-MYRF-1:577 robustly bound the FLAG beads (unpublished data). Thus, an intact ICA domain is essential for the formation of the N-terminal trimer.

As bacteriophage ICA domains require the completion of folding and trimerization as a prerequisite to the auto-cleavage reaction [20],[36], we suspected that full-length MYRF should also homo-oligomerize. A test of full-length MYRF, obtained by using a catalytic residue mutant (K583A), confirmed its homo-oligomerization (Figure 4B). Likewise, full-length MYRF obtained by a leucine zipper mutant (L683A) still homo-oligomerized (Figure 4B), in spite of defective auto-processing (Figure 3C). Thus, auto-processing of MYRF apparently requires both trimerization and proper formation of the leucine zipper that includes L683. Notably, the N-terminal fragment generated from the auto-processing of MYRF-1:756 (Figure 4A) also formed a homo-oligomer (Figure 4B), consistent with functional autonomy of the ICA domain.

Upon auto-processing, the N-terminal trimer translocates to the nucleus. To test the roles of the predicted NLSs (K_245_KRK_248_ and K_482_KGK_485_) in nuclear translocation, we examined the effects of deleting the NLSs on subcellular localization. When either single NLS was deleted (3F-MYRFΔNLS1 or 3F-MYRFΔNLS2), nuclear translocation of the N-terminal trimer was only partially blocked (Figure 4C). Deletion of both NLSs blocked MYRF nuclear translocation to a greater extent (Figure 4C), indicating that both NLSs contribute to the nuclear translocation of the N-terminal trimer.

### Auto-Processing Is Essential for the Transcriptional Activity of MYRF

Once generated as a type-II membrane protein, MYRF is auto-processed into two independent fragments (Figure 3B). The N-terminal trimer enters the nucleus where it is likely to function as a transcription factor, while the C-terminal homo-oligomer remains in the ER, where its function is unknown. In order to assay the transcriptional roles of MYRF, we first identified transcriptional targets of MYRF by performing next-generation RNA sequencing of HeLa cells that were transfected with wild-type MYRF and a catalytic residue mutant. Among genes differentially expressed between the two samples (Table S1), we confirmed *Endothelin 2* (*Edn2*) as a transcriptional target of MYRF in HeLa cells, and thus could use its expression levels measured by quantitative real-time polymerase-chain-reaction (qRT-PCR) as a readout of the transcriptional activity of various MYRF constructs.

**Figure 5:**
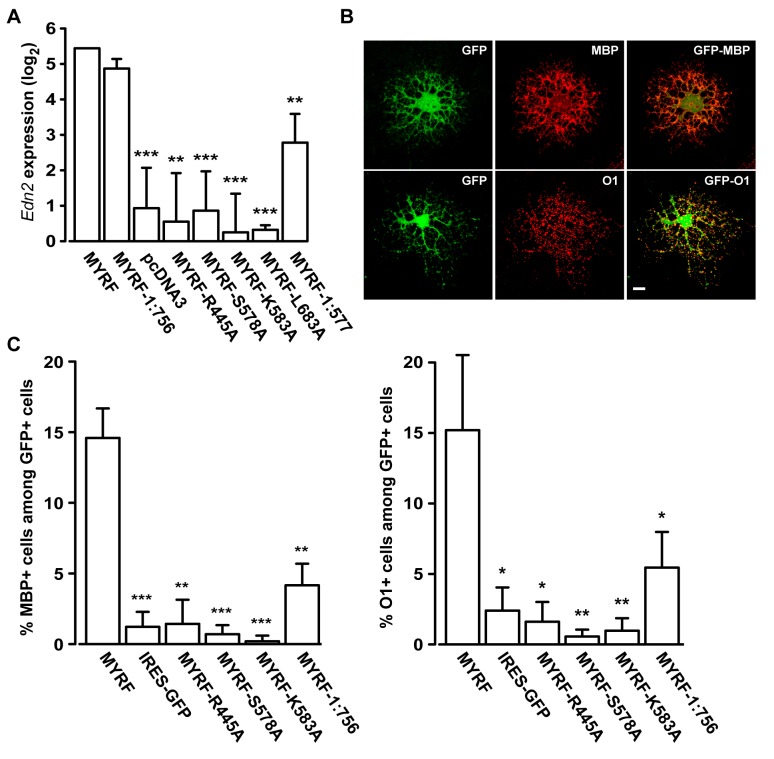
Auto-processing is essential for the functions of MYRF. (A) The transcriptional activity of various MYRF constructs was estimated by their ability to activate the transcription of *Edn2* in HeLa cells. Values are means ± SEM. (B) Examples of transfected CG4 cells that matured to express MBP or O1. (C) Quantification of the proportion of transfected CG4 cells expressing MBP or O1. Values are means ± SEM. *p<0.05, **p<0.01, and ***p<0.001. Scale bar, 10 µm.

Using this assay, we confirmed that auto-processing of MYRF is required for the transcriptional activity of MYRF. Figure 5A shows that the expression level of *Edn2* was about 20-fold higher when HeLa cells were transfected with MYRF compared to the empty vector control (pcDNA3). The transcriptional activation of *Edn2* by MYRF is most likely due to the direct binding of MYRF to DNA because mutation of R445, a strictly conserved residue essential for the direct binding of MrfA to DNA [37], to alanine ablated its transcriptional effects (Figure 5A). Mutation of R445 to alanine did not affect the auto-processing and localization of MYRF (Figure S5). Blocking the auto-processing of MYRF, by mutating either catalytic residues (S578A and K583A) or a structurally important residue (L683A), abrogated the transcriptional effects of MYRF on *Edn2* (Figure 5A).

To test whether the N-terminal trimer is sufficient for this transcriptional activation, we assayed a construct truncated before the TM domain at L756 (MYRF-1:756) for transcriptional activity. MYRF-1:756 properly homo-oligomerizes (Figure 4B) and is normally processed (Figure 3F). Figure 5A shows that MYRF-1:756 is as competent as full-length MYRF in activating the transcription of *Edn2*, demonstrating that the N-terminal trimer is sufficient for the transcriptional activity of MYRF; notably, the C-terminal homo-oligomer does not significantly contribute to this activity. Although homo-trimeric transcription factors are not common, there is a well-known precedent, heat shock factor 1 [38].

Trimerization also appears to be necessary for full transcriptional effects, as we observed a construct directly encoding the N-terminal fragment of MYRF (MYRF-1:577, Figure 4A) to be only partially functional (Figure 5A); this construct fails to form a trimer (Figure 4B). On the other hand, our observation that MYRF-1:577 is still partially functional is in excellent agreement with a recent demonstration that monomeric MrfA can still bind DNA *in vitro*, although it appears to function as a trimer *in vivo* [37].

### Auto-Processing Is Essential for MYRF to Promote OL Maturation

Given the well-characterized role of Myrf (the mouse ortholog) in OL maturation [17], we examined the functional consequences of MYRF auto-processing on the maturation of CG4 cells. Although the CG4 cell line is widely understood not to be a good model of myelination, it may be used as a model for early OL differentiation. We counted the fraction of transfected CG4 cells that had matured to express myelin basic protein (MBP) or O1, two known OL maturation markers (Figure 5B). MYRF significantly promoted the maturation of CG4 cells: 15% of transfected CG4 cells matured to express MBP when transfected with a vector containing both MYRF and GFP (MYRF, Figure 5C), as compared to less than 2% of cells transfected with a control vector expressing only GFP (IRES-GFP). Consistent with the RT-PCR analysis, the mutation of R445 to alanine abrogated the effects of MYRF on CG4 cell maturation (MYRF-R445A, Figure 5C), suggesting that MYRF directly binds DNA to activate transcription for OL maturation. Auto-processing mutants of MYRF (MYRF-S578A and MYRF-K583A) similarly failed to promote CG4 cell maturation (Figure 5C), indicating that correct processing is required. To test if the N-terminal trimer generated by auto-processing is sufficient for OL maturation, we employed a construct truncated before the TM domain at L756 (MYRF-1:756, Figure 4A). Notably, MYRF-1:756 was much less competent compared to wild-type MYRF in promoting maturation (Figure 5C), in spite of being normally processed (Figure 3F), homo-oligomerizing (Figure 4B), and activating *Edn2* expression similarly to wild-type MYRF (Figure 5A) in HeLa cells, suggesting a potential role for the C-terminal domain in OL maturation. Overall, our data confirm that auto-processing is essential for MYRF to promote OL maturation. They also suggest that the maturation of OLs might require both the transcription factor function of the N-terminal trimer and the unknown function of the C-terminal homo-oligomer in the ER.

## Discussion

RIP- and RUP-activated MBTFs are widely observed across organisms, spanning both eukaryotes and prokaryotes. MBTFs are recognized as increasingly common regulatory mechanisms in plants, with many plant MBTFs playing important roles in stress responses and development [39]–[43]. NTM1 (NAC with TM motif1), for example, regulates cell division and growth in *Arabidopsis* [44]. New examples of MBTFs have also been identified for bacteria [45]–[49]. In fact, ToxR of *Vibrio cholerae* was the first known MBTF [50], although it is still unclear whether ToxR requires a proteolytic activation step to exert transcriptional effects. Thus, it is likely that many MBTFs remain to be found, and an open question is what other activation mechanisms may be employed. MYRF reveals one such previously unknown activation mechanism.

### MYRF Is a Founding Member of a New Family of MBTFs That Are Auto-Processed Into Two Independent Trimers by an ICA Domain

**Figure 6:**
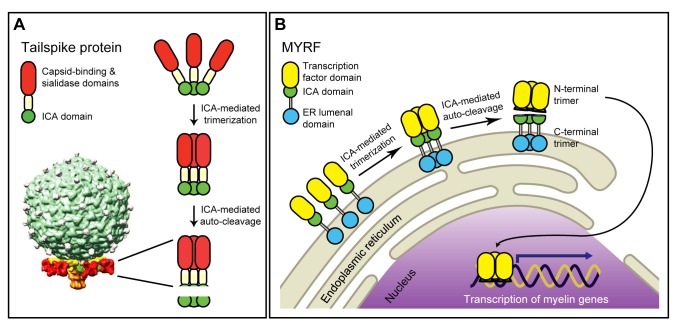
The ICA domain catalyzes trimerization-dependent auto-proteolysis in entirely distinct protein and cellular contexts. (A) In K1F bacteriophage, the C-terminal ICA domain within each tailspike endosialidase auto-catalytically removes itself following tailspike trimerization, guiding maturation of the six tailspikes surrounding the phage tail (shown for phage K1E, adapted from [55]). (B) Once generated as a type-II membrane protein, the ICA domain is thought to induce the trimerization of MYRF, upon which it cleaves itself, generating two independent trimers. The N-terminal trimer translocates to the nucleus and activates the transcription of myelin genes by direct DNA binding. The transcriptional role of the N-terminal trimer serves to promote the terminal differentiation of OLs, likely aided by an as-yet-unknown function of the C-terminal trimer that remains in the ER.

We show that MYRF is a MBTF that is auto-processed by its ICA domain into two independent homo-oligomers (Figure 6B). The N-terminal trimer, containing a largely disordered protein segment and the Ndt80 DNA-binding domain, is released from the membrane and translocates to the nucleus to regulate gene expression. The disordered N-terminal protein segment presumably functions as a transactivation domain because partial deletions in this region render MYRF nonfunctional in terms of its transcriptional activation of *Edn2*, although auto-processing and localization are not affected (unpublished data). The N-terminal trimer is both necessary and sufficient for the transcriptional activity of MYRF. The C-terminal homo-oligomer remains in the ER, and may perform an important function there. Our functional assays show that auto-processing is essential for MYRF both to activate transcription and to promote OL maturation.

Importantly, this mechanism is probably conserved across all MYRF family members that include MYRFL (a paralog of MYRF), as all members—found across the animal kingdom, including vertebrates, insects, nematodes, amoeba, and tunicates—are characterized by the domain arrangement shown in Figure 3B. Residues shown to be essential for the direct DNA-binding of the Ndt80 DNA-binding domain [18], including MYRF R445 and R469, are strictly conserved across the family, as are key residues of the ICA domain, such as S578, D579, K583, G626, and I687, and the presence of the leucine zipper. The ICA domain is invariantly followed by a relatively well-conserved TM domain and a poorly conserved ER lumenal domain.

### Does the ICA Domain Function as a Chaperone?

As an intramolecular chaperone, the ICA domain is known to facilitate the trimerization and folding of protein sequences that lie N-terminal to it [36], and it has been best characterized for bacteriophage endosialidases [19]–[20]. Without the ICA domain, the endosialidases apparently fail to fold properly, let alone forming a trimer. For the N-terminal fragment of MYRF, however, the role of the ICA domain seems limited to trimerization. Even though MYRF-1:577 failed to form a trimer (Figure 4B), it did activate the transcription of *Edn2*, albeit to a lesser degree compared to its trimeric counterpart. Also, the presumably monomeric form of MrfA bound DNA [37]. These results suggest that while the ICA domain has a clear role in trimerization and auto-catalysis, its role in the proper folding of the N-terminal fragment of MYRF may be less critical than for the folding of bacteriophage endosialidases. Consistent with this, sequence alignments show that an extended loop of the bacteriophage K1F ICA domain that comprises T977–H1026, shown to be essential for the folding of bacteriophage endosialidases [20], is missing in most other ICA domains, including those of MYRF and its orthologs (Figure S3A).

From a structural perspective, the ICA domain achieves trimerization by chaperoning a triple β-helix fold [36]. Triple β-helix folds are often associated with very stable trimeric structures such as viral tailspike or fiber proteins, and the bacteriophage K1F endosialidase—whose trimerization and folding are mediated by its ICA domain—is known to form a trimeric structure that is resistant to SDS [19]. An interesting question is whether the N-terminal trimer of MYRF also involves a triple β-helix fold. Figure S3D shows that the region compassing residues W536-P577, where a triple β-helix fold is expected, is indeed well conserved. In PQN-47, a mutation of the glycine residue equivalent to G566 of MYRF renders PQN-47 nonfunctional [51]. The evidence therefore suggests that the N-terminal trimer of MYRF could in principle maintain its trimeric state by having a triple β-helix fold at its C-terminus.

### Why Are MYRF and Its Orthologs Membrane-Bound?

The auto-processing of MYRF appears to be constitutive, which stands in stark contrast to the highly regulated processing of such factors as SREBP [4], ATF6 [5], Notch [9], and STP23 [1]. In fact, we observed normal processing of MYRF in all the systems that we tried, including HEK293 cells, fibroblasts, human umbilical vein endothelial cells, and frog embryos (unpublished data), in addition to the budding yeast and *E. coli*. Nevertheless, we do not exclude the possibility that the auto-processing of MYRF can in principle be regulated by some mechanism. The apparently constitutive processing of MYRF presents a puzzle, as our data suggest that the processing is not a key step by which MYRF might be externally regulated. Indeed, Myrf (the mouse ortholog) has been shown to be regulated mainly at the transcriptional level [17]. Moreover, another recent study indicates that Myrf is continuously needed throughout adulthood to maintain myelin, consistent with a constitutive process [52]. Why, then, are MYRF and its orthologs MBTFs? While we have demonstrated that the mechanism clearly supports the proper assembly of the N-terminal transcription factor trimer, we speculate that this might additionally represent a mechanism by which the generation of two functionally independent trimers can be mandatorily coupled to coordinate regulation of nuclear and ER processes.

Several pieces of circumstantial evidence support this speculation: First, PQN-47, the *C. elegans* ortholog of MYRF, has recently been implicated in the regulation of molting—a process involving extensive secretion [51]. Notably, an intact ER lumenal domain, localized outside the nucleus, was shown to be critical for PQN-47's regulation of molting. Second, our CG4 maturation assay shows that the N-terminal trimer alone is not as competent as full-length MYRF in promoting the maturation of CG4 cells, suggesting that the unknown function of the C-terminal homo-oligomer may be as essential for OL maturation as the transcriptional function of the N-terminal trimer. Notably, the physiological processes and genes to which MYRF and its orthologs have been linked all involve the secretory pathway. *EcmA* (an endogenous target gene of MrfA [37]) is a secreted protein. Myelination, in which Myrf plays an essential role, places heavy demands upon the secretory pathway, as does molting, for which PQN-47 is critical [51]. Finally, a meta-analysis of microarray data in the Gene Expression Omnibus database [53] indicates that MYRF is significantly expressed in secretory tissues including stomach and lung (unpublished data). We speculate that in the context of OL differentiation, while the N-terminal trimer acts in the nucleus to stimulate production of myelin components, the C-terminal homo-oligomer either coordinates their orderly passage through the secretory pathway or functions as part of the UPR pathway to prepare the ER for the increased flux of myelin components. In the future, it will be interesting to explore whether MYRF is truly a dual-functional protein and what function the C-terminal homo-oligomer performs in the ER for OL maturation.

### Reconciling Conflicting Reports on Myrf and Its Orthologs

In fact, the recognition of this protein family as MBTFs serves to reconcile apparently contradictory, but likely correct, findings in the prior literature. The report on Myrf ascribed its role to a master transcriptional regulator for OL maturation and CNS myelination [17]. A subsequent study on PQN-47 questioned this role for Myrf, mainly because a PQN-47::GFP translational fusion protein localized outside the nucleus in *C. elegans* [51]. Based on molting phenotypes, the PQN-47 study concluded that PQN-47 (and Myrf, by implication) might play an important role in the secretory pathway. On the other hand, a recent study in *Dictyostelium* showed that the DNA-binding domain of MrfA endogenously localizes to the nucleus and binds DNA directly, supporting the conclusion of the report on Myrf [37].

Our finding that MYRF is a MBTF that is auto-processed into two independent homo-oligomers entirely resolves these seemingly conflicting reports: Depending on the location of the epitope tag and whether auto-processing is blocked or not, MYRF and its orthologs can exhibit either nuclear or ER localization, as appropriate; each of these prior studies is consistent with this interpretation. Taken together, MYRF and its orthologs represent a new class of MBTFs that require auto-processing to function in gene transcription and likely also play important roles beyond transcription, including in secretion.

## Materials and Methods

### Constructs, Cell Culture, and Transient Transfection

The MYRF cDNA was purchased from Open Biosystems (the 1111-amino-acid-long isoform [CCDS ID: 31579 and RefSeq ID: NP_037411]). HeLa cells were cultured in Dulbecco's modified Eagle's medium supplemented with 10% fetal bovine serum. Cells were maintained in a humidified 5% CO_2_ incubator at 37°C. Transient transfection was performed using either FuGENE HD or Lipofectamine 2000.

### Immunoprecipitation

Cells grown on 150 mm culture dishes were rinsed once with PBS, and 500 µL of 2× Cell Lysis Buffer (Cell Signaling) was directly added to the cell layer. Cell lysates were sonicated and then spun down at 14,000× *g* for 10 min at 4°C. The supernatant was mixed with FLAG antibody-coated beads (Sigma) and incubated for 2 h at 4°C on a rotating plate. The mix was spun down at 7,500× *g* for 30 s to separate supernatant (“Sup”) from pellet (“Bead”) fractions.

### Immunoblotting

Cells were rinsed once with PBS and then lysed directly in wells with 1× Laemmli Sample Buffer (Bio-Rad). Cell lysates were boiled at 95°C for 5 min. Upon SDS-PAGE, the proteins were transferred to PVDF and probed with primary and horseradish peroxidase (HRP)-conjugated secondary antibodies. The following dilutions were used for immunoblotting: mouse anti-FLAG (1∶1000, Sigma), rabbit anti-c-Myc (1∶250, Santa Cruz Biotechnology), goat anti-actin (1∶400, Santa Cruz Biotechnology), rabbit anti-calnexin (1∶400, Santa Cruz Biotechnology), goat anti-calnexin (1∶400, Santa Cruz Biotechnology), goat anti-mouse HRP-conjugated (1∶10,000, Santa Cruz Biotechnology), mouse anti-FLAG HRP-conjugated (1∶1,000, Sigma), and mouse anti-c-Myc HRP-conjugated (1∶400, Santa Cruz Biotechnology).

### Immunofluorescence

Cells cultured in glass bottom six-well plates (In Vitro Scientific) were fixed with 4% formaldehyde, permeabilized in cold 100% methanol or 0.1% Triton X-100, blocked with 1% BSA in 1× PBS with 0.05% Tween, and incubated with primary antibody diluted in blocking buffer at 4°C overnight, followed by incubation with fluorochrome-conjugated secondary antibody. Nuclei were stained with Hoechst 33342 (Invitrogen). Fluorescence was visualized with a Nikon Eclipse TE2000-E fitted with a Plan Apo VC 100×/1.40 oil objective and a digital camera (Cascade II 512; Photometrics) controlled by the NIS Elements software (AR 3.0). To selectively permeabilize plasma and ER membranes, 25 µg/ml digitonin was used to treat cells for 5 min on ice, followed by fixation with 4% formaldehyde. Rhodamine-conjugated donkey anti-goat IgG was from Santa Cruz Biotechnology. Alexa Fluor 594 goat anti-mouse or rabbit IgG and Alexa Fluor 488 goat anti-rabbit IgG were from Invitrogen.

### Protein Expression and Purification in *E. coli*

The truncated MYRF (MYRF-319:708) was inserted into pET52b (Novagen) between BamHI and SacI to generate pET52b-StrepII-MYRF-319:708-10xHis. This plasmid was transformed into BL21 Star (DE3) pLysS *E. coli* (Invitrogen). Cells were cultured at 37°C to OD_600 nm_ 0.4–0.6 in LB and protein expression was induced by 0.5 mM IPTG at 16°C for 16–18 h. Cells were collected and lysed by sonication in lysis buffer (20 mM Tris pH 8.0, 500 mM NaCl, 10 mM β-mercaptoethanol, 10% glycerol). The lysate was clarified by centrifugation at 15,000× g for 30 min at 4°C and the supernatant was loaded onto a Ni-NTA column (Qiagen). The flow-through was loaded onto a Strep-Tactin chromatography column (IBA) to affinity purify the N-terminal fragment of MYRF-319:708 according to the manufacturer's purification protocol. The eluted protein was dialyzed in dialysis buffer (20 mM HEPES pH 7.5, 50 mM NaCl, 10 mM β-mercaptoethanol, 10% glycerol) and concentrated to about 2 mg/ml. The concentrated protein was analyzed by Superdex 200 10/300 GL gel filtration column chromatography.

### qRT-PCR

RNA was extracted from HeLa cells using Trizol (Invitrogen). cDNA was generated using SuperScript First-Strand Synthesis System for RT-PCR (Invitrogen). qRT-PCR was performed using PowerSYBR Green PCR Master Mix (Invitrogen) and ABI ViiA 7 Real-Time PCR System. Primer sequences are available in Table S2.

### CG4 Maturation Assay

CG4 cells were maintained in GM [70% of CG4 growth medium (Dulbecco's modified Eagle's medium, 5 µg/ml transferrin, 100 µM putrescine, 20 nM progesterone, 30 nM selenium, 10 ng/ml biotin, and 5 µg/ml insulin) supplemented with 30% of the same medium conditioned by B104 cells]. For maturation assays, CG4 cells were plated on glass bottom six-well plates coated with poly-L-ornithine. 0.4 µg of plasmid DNA was transfected using Lipofectamine 2000 (Invitrogen) for 4 h. After transfection, GM was replaced by DM (CG4 growth medium supplemented with 1% FBS, 40 ng/ml triiodothyronine). CG4 cells were maintained in DM for 4 d before immunostaining for cell counting. Primary antibodies used were 1∶500 mouse anti-O1 (Millipore) and 1∶500 rat-anti-MBP (Millipore). For each sample, cells of at least 50 random fields were counted in a blind fashion.

## Supporting Information

Figure S1 Control IF experiments confirmed that MYRF is generated as a membrane protein. (A) Membrane topology prediction results for MYRF (left) and Myrf (right) from the TOPCONS server [15]. (B) IF images of 3F-MYRF, MYRF-3F, and MYRFΔTM-3F in HeLa cells. (C) IF images of GFP-MYRF and MYRF-GFP in CG4 cells. (D) IF image of 3F-MYRF-GFP in CG4 cells. Scale bars, 10 µm.
(TIF)

Figure S2 Disappearance of the middle band at ∼120 kDa upon MG132 treatment (A) and control experiments for selective membrane permeation with digitonin (B). (A) HeLa cells were transfected with 3F-MYRF and then treated with MG132, a proteasome inhibitor. The middle band disappeared upon MG132 treatment, suggesting that it represents a proteasome degradation intermediate, presumably caused by overexpression. This possibility was corroborated by a control experiment with p60Tth, the NFκB p105 construct whose processing is known to be mediated by the proteasome [56]. (B) Control experiments testing the selective permeation of the plasma membrane by digitonin. When cells were selectively permeated by digitonin, a calnexin antibody targeting an epitope inside the ER lumen did not yield IF signals. Yet when cells were indiscriminately permeated by Triton X-100, it gave strong IF signals. An antibody targeting an epitope in the cytoplasmic segment of calnexin gave IF signals for both digitonin and Triton X-100. Scale bar, 10 µm.
(TIF)

Figure S3 The ICA domain autonomously mediates the proteolytic processing of MYRF. (A) Sequence alignment between the ICA domain of bacteriophage K1F endosialidase and the portion of MYRF that lies between its DNA-binding and TM domains, as generated by the HHpred server [34]. (B) L683 and L690 were predicted to form a leucine zipper. Mutation of these residues to alanine disrupted the processing of MYRF. IF images confirmed their exclusion from the nucleus. (C) Mapping of the amino acid sequence of MYRF onto the ICA domain of bacteriophage K1F endosialidase (PDB ID: 3GW6), based on the sequence alignment shown in panel A, indicated the positions that K596, S599, and L679 of MYRF would occupy. Since these three residues were all predicted to point outward, they were not expected to be critical for either catalytic or structural roles. (D) Multiple sequence alignment of MYRF and its orthologs generated by ClustalW [54]. Shown are the DNA-binding domain (blue broken double line), the ICA domain (red broken double line), and the TM domain (black broken double line). Scale bar, 10 µm.
(TIF)

Figure S4 The N-terminal fragment of MYRF forms a trimer. MYRF-319:708 was expressed in *E. coli* with N-terminal StrepII tag and C-terminal His tag. The N-terminal fragment StrepII-MYRF-319:577 from the auto-processing of StrepII-MYRF-319:708-10xHis was purified by Strep-Tactin affinity chromatography followed by size exclusion chromatography. The elution profiles of molecular weight standards and StrepII-MYRF-319:577 are shown in panels A and B, respectively. The peak for StrepII-MYRF-319:577 was confirmed by SDS-PAGE (the insert in panel B). The molecular weight of StrepII-MYRF-319:577 was determined to be 89 kDa by comparison with a standard curve. Theoretical molecular weights for a monomer, dimer, and trimer are 30 kDa, 60 kDa, and 90 kDa, respectively.
(TIF)

Figure S5 R445A mutation does not affect the proteolytic processing and localization of MYRF. (A) Western blot showed that 3F-MYRF-R445A is normally processed. (B) IF images showed that the N-terminal fragment of 3F-MYRF-R445A is localized in the nucleus. Scale bar, 10 µm.
(TIF)

Table S1 Ten most differentially expressed genes between HeLa cells that were transfected with wild-type MYRF and the catalytic mutant S578A.
(DOCX)

Table S2 Primer sequences for qRT-PCR.
(DOCX)

## Abbreviations

CLX: calnexin
ER: endoplasmic reticulum
GFP: green fluorescent protein
ICA: intramolecular chaperone auto-processed
IF: immunofluorescence
MBP: myelin basic protein
MBTF: membrane-bound transcription factor
NLS: nuclear localization signal
OL: oligodendrocyte
RIP: regulated intramembrane proteolysis
RUP: regulated ubiquitin/proteasome-dependent processing
TM: transmembrane
