# Supplementary figures and images for: A Bacteriophage Tailspike Domain Promotes Self-Cleavage of a Human Membrane-Bound Transcription Factor, the Myelin Regulatory Factor MYRF
Source: PLoS Biol. 2013 Aug 13;11(8):e1001624. doi: 10.1371/journal.pbio.1001624 (PMC3742443; doi:10.1371/journal.pbio.1001624)

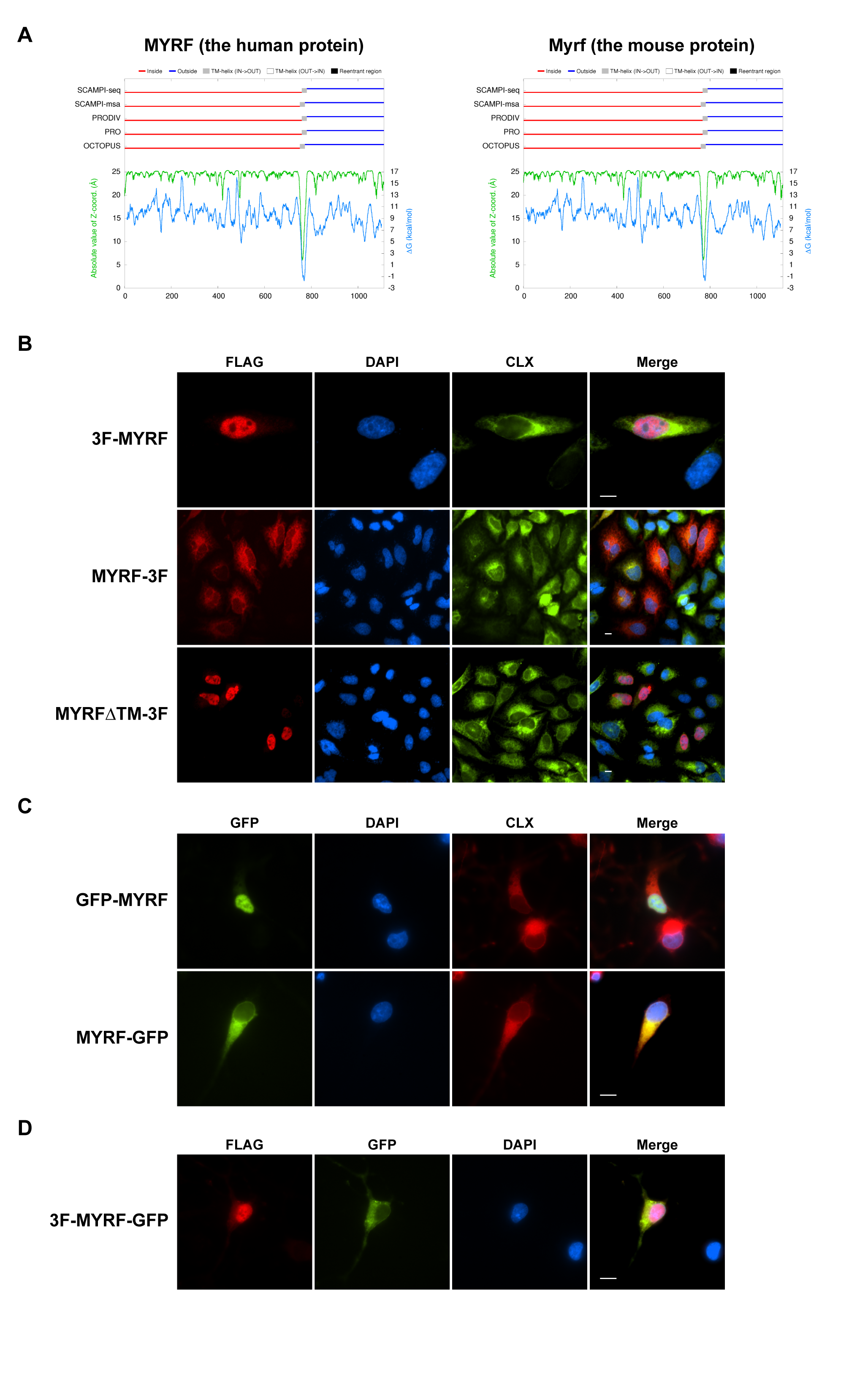

Supplement: Figure S1 — Control IF experiments confirmed that MYRF is generated as a membrane protein. (A) Membrane topology prediction results for MYRF (left) and Myrf (right) from the TOPCONS server [15]. (B) IF images of 3F-MYRF, MYRF-3F, and MYRFΔTM-3F in HeLa cells. (C) IF images of GFP-MYRF and MYRF-GFP in CG4 cells. (D) IF image of 3F-MYRF-GFP in CG4 cells. Scale bars, 10 µm. (TIF) [file pbio.1001624.s001.tif]

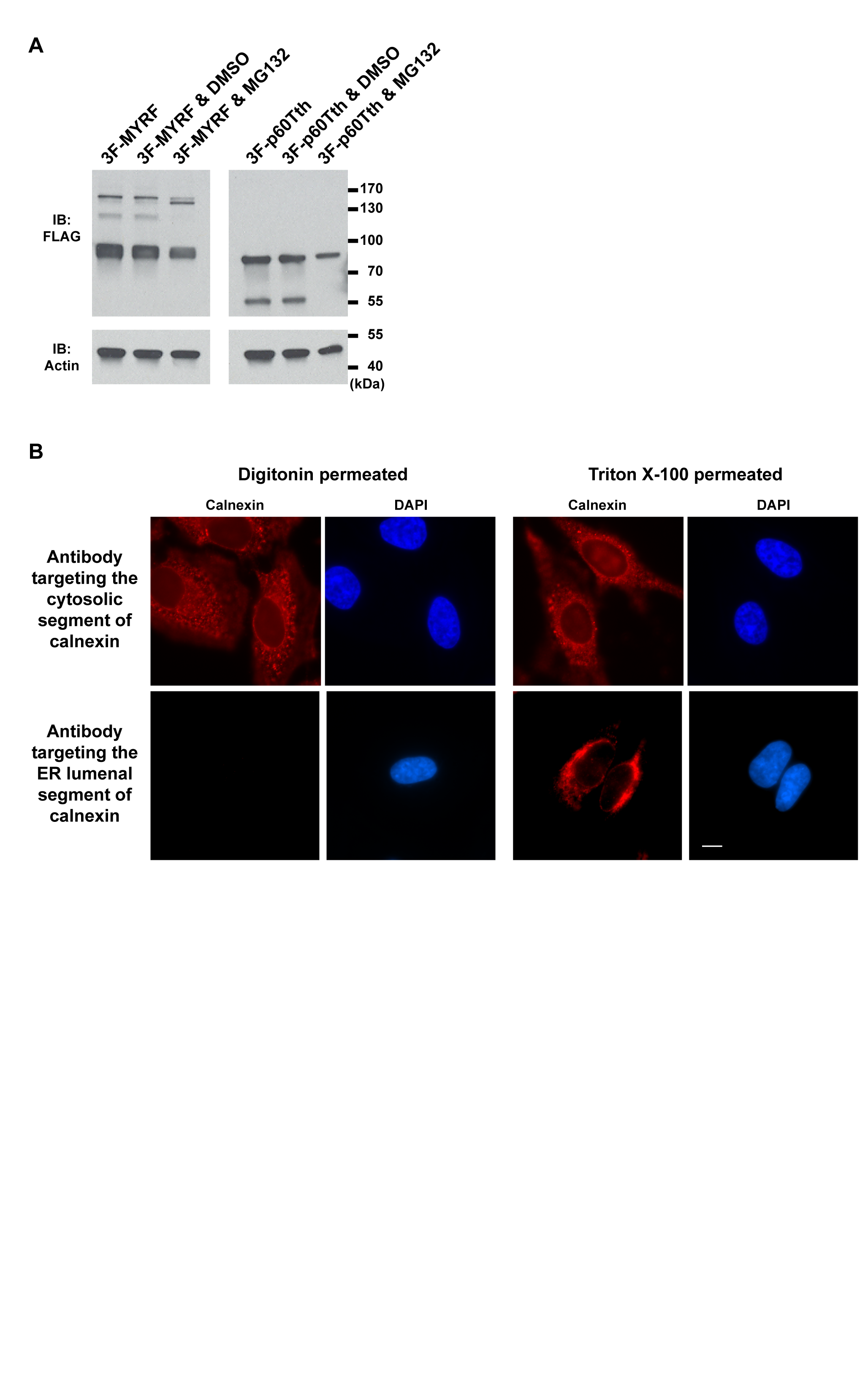

Supplement: Figure S2 — Disappearance of the middle band at ∼120 kDa upon MG132 treatment (A) and control experiments for selective membrane permeation with digitonin (B). (A) HeLa cells were transfected with 3F-MYRF and then treated with MG132, a proteasome inhibitor. The middle band disappeared upon MG132 treatment, suggesting that it represents a proteasome degradation intermediate, presumably caused by overexpression. This possibility was corroborated by a control experiment with p60Tth, the NFκB p105 construct whose processing is known to be mediated by the proteasome [56]. (B) Control experiments testing the selective permeation of the plasma membrane by digitonin. When cells were selectively permeated by digitonin, a calnexin antibody targeting an epitope inside the ER lumen did not yield IF signals. Yet when cells were indiscriminately permeated by Triton X-100, it gave strong IF signals. An antibody targeting an epitope in the cytoplasmic segment of calnexin gave IF signals for both digitonin and Triton X-100. Scale bar, 10 µm. (TIF) [file pbio.1001624.s002.tif]

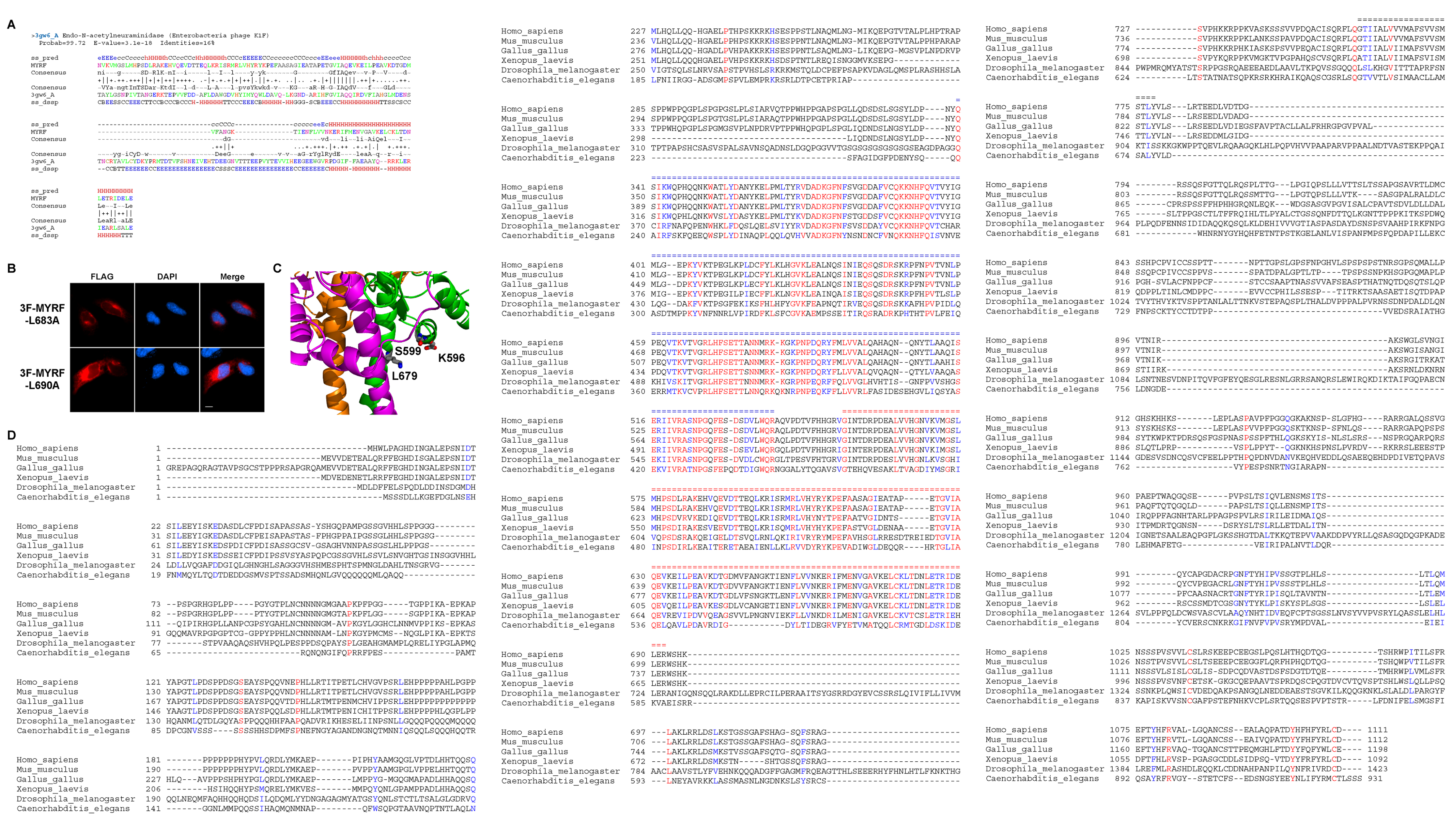

Supplement: Figure S3 — The ICA domain autonomously mediates the proteolytic processing of MYRF. (A) Sequence alignment between the ICA domain of bacteriophage K1F endosialidase and the portion of MYRF that lies between its DNA-binding and TM domains, as generated by the HHpred server [34]. (B) L683 and L690 were predicted to form a leucine zipper. Mutation of these residues to alanine disrupted the processing of MYRF. IF images confirmed their exclusion from the nucleus. (C) Mapping of the amino acid sequence of MYRF onto the ICA domain of bacteriophage K1F endosialidase (PDB ID: 3GW6), based on the sequence alignment shown in panel A, indicated the positions that K596, S599, and L679 of MYRF would occupy. Since these three residues were all predicted to point outward, they were not expected to be critical for either catalytic or structural roles. (D) Multiple sequence alignment of MYRF and its orthologs generated by ClustalW [54]. Shown are the DNA-binding domain (blue broken double line), the ICA domain (red broken double line), and the TM domain (black broken double line). Scale bar, 10 µm. (TIF) [file pbio.1001624.s003.tif]

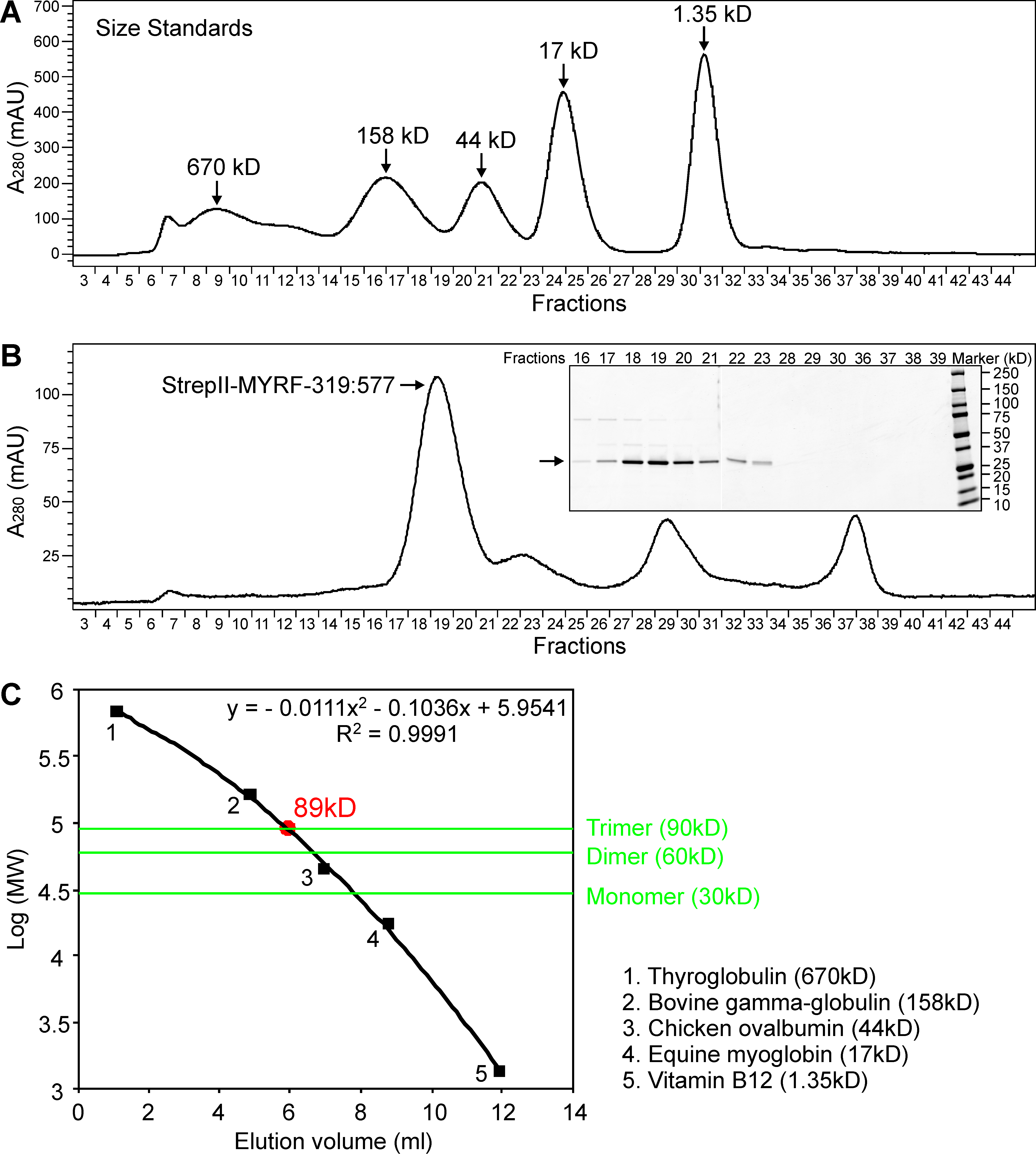

Supplement: Figure S4 — The N-terminal fragment of MYRF forms a trimer. MYRF-319:708 was expressed in E. coli with N-terminal StrepII tag and C-terminal His tag. The N-terminal fragment StrepII-MYRF-319:577 from the auto-processing of StrepII-MYRF-319:708-10xHis was purified by Strep-Tactin affinity chromatography followed by size exclusion chromatography. The elution profiles of molecular weight standards and StrepII-MYRF-319:577 are shown in panels A and B, respectively. The peak for StrepII-MYRF-319:577 was confirmed by SDS-PAGE (the insert in panel B). The molecular weight of StrepII-MYRF-319:577 was determined to be 89 kDa by comparison with a standard curve. Theoretical molecular weights for a monomer, dimer, and trimer are 30 kDa, 60 kDa, and 90 kDa, respectively. (TIF) [file pbio.1001624.s004.tif]

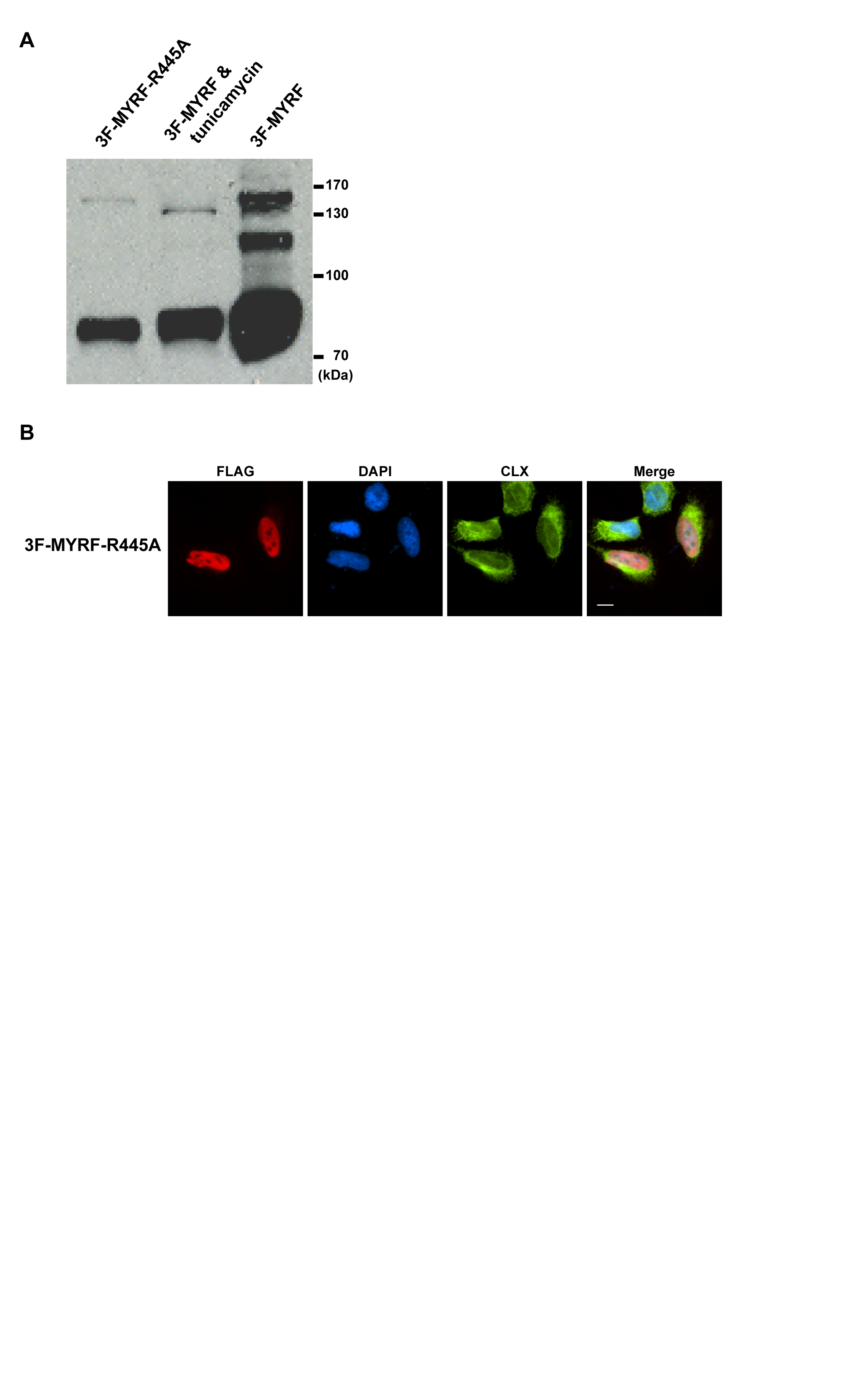

Supplement: Figure S5 — R445A mutation does not affect the proteolytic processing and localization of MYRF. (A) Western blot showed that 3F-MYRF-R445A is normally processed. (B) IF images showed that the N-terminal fragment of 3F-MYRF-R445A is localized in the nucleus. Scale bar, 10 µm. (TIF) [file pbio.1001624.s005.tif]
