# Supplementary material for: A Bacteriophage Tailspike Domain Promotes Self-Cleavage of a Human Membrane-Bound Transcription Factor, the Myelin Regulatory Factor MYRF
Source: PLoS Biol. 2013 Aug 13;11(8):e1001624. doi: 10.1371/journal.pbio.1001624 (PMC3742443; doi:10.1371/journal.pbio.1001624)
Supplement: Table S1 — Ten most differentially expressed genes between HeLa cells that were transfected with wild-type MYRF and the catalytic mutant S578A. (DOCX) [file pbio.1001624.s006.docx]

**Table S1. Ten most differentially expressed genes between HeLa cells that were transfected with wild-type MYRF and the catalytic mutant S578A.**

| *Edn2* | *Furin* | *Nacc2* | *Tmem107* | *Aldh1a3* |
| --- | --- | --- | --- | --- |
| *Smarcd3* | *Metrnl* | *Tnfaip2* | *Cdkn1c* | *Khdrbs3* |
