# Supplementary material for: A Bacteriophage Tailspike Domain Promotes Self-Cleavage of a Human Membrane-Bound Transcription Factor, the Myelin Regulatory Factor MYRF
Source: PLoS Biol. 2013 Aug 13;11(8):e1001624. doi: 10.1371/journal.pbio.1001624 (PMC3742443; doi:10.1371/journal.pbio.1001624)
Supplement: Table S2 — Primer sequences for qRT-PCR. (DOCX) [file pbio.1001624.s007.docx]

**Table S2. Primer sequences for qRT-PCR**

|  | Forward primer | Backward primer |
| --- | --- | --- |
| *GAPDH* | CTTTGTCAAGCTCATTTCCTG | TCTTCCTCTTGTGCTCTTGC |
| *MYRF* | AGCCCAAGGCTCCCTATG | AGGAGGTGGGGCTCATTC |
| *Edn2* | CTCGACAAGGAGTGCGTCTA | AGGCCGTAAGGAGCTGTCTG |
